# Blood Reference Intervals for Antillean Manatees (*Trichechus manatus manatus*) from Puerto Rico

**DOI:** 10.1155/2022/4305838

**Published:** 2022-10-18

**Authors:** Antonio A. Mignucci-Giannoni, Mayela M. Alsina-Guerrero

**Affiliations:** ^1^Caribbean Manatee Conservation Center, Inter American University of Puerto Rico, Bayamón, PR, USA; ^2^Center for Conservation Medicine and Ecosystem Health, Ross University School of Veterinary Medicine, Basseterre, Saint Kitts and Nevis; ^3^Georgia Aquarium, Atlanta, Georgia, USA

## Abstract

Antillean manatees (*Trichechus manatus manatus*) are endangered throughout the southwestern Gulf of Mexico, the Caribbean coast of Central and South America, the Greater Antilles, and the northeastern coast of South America to Brazil. Establishing blood reference intervals is essential as a tool in classifying health status, diagnosing, establishing treatment regimens, and monitoring the progress of a disease in rescued manatees. We collected blood samples from 44 free-ranging and 26 rescued manatees from Puerto Rico between 1992 and 2020 for hematology and blood chemistry analysis. We obtained values for white blood cell count and red blood cell count, hemoglobin, hematocrit, platelet, mean cell volume, mean cell hemoglobin, mean cell hemoglobin concentration, and red cell distribution width. A manual leukocyte differential allowed for the evaluation of different cell types. In addition, we performed a comprehensive metabolic panel on serum samples. These analytes were grouped based on six physiologic processes: liver-associated enzymes and pigments; muscle-associated enzymes; kidney-associated compounds and products; sugars, lipids, and pancreatic-associated enzymes; proteins; and electrolytes. For every parameter, summary statistics of values were calculated on all the samples. Reference ranges were determined as ±1 standard deviation around the mean. An unpaired two-sample *T*-test was done comparing males versus females and adults versus calves for any significant differences (*p* ≤ 0.05). We establish the reference intervals of hematology and blood chemistry for the population of Antillean manatees in Puerto Rico and compare them with those established for manatees from Belize, Brazil, Florida, Guyana, and Mexico.

## 1. Introduction

The Antillean manatee (*Trichechus manatus manatus*) is a subspecies of the West Indian manatee commonly found throughout the southwestern Gulf of Mexico, the Caribbean coast of Central and South America, the Greater Antilles, and the northeastern coast of South America to Brazil [1, 2]. It is an endangered marine mammal [2] protected by federal and local laws in their respective countries of origin. Its vulnerability is primarily due to anthropogenic causes of direct hunting, habitat degradation, and human encroachment, resulting in a decrease in population and continued threats to the species' survival [3, 4]. As a result, conservation programs have been established to assist in the recovery of the populations, particularly addressing the rescue, treatment, rehabilitation, and release of orphaned calves and ill or injured manatees [5].

Establishing blood reference intervals is essential as a tool in classifying health status, diagnosing, determining treatment regimens, and monitoring the progress of a disease in rescued manatees [6]. However, blood reference ranges or values can differ among populations of the same species and vary according to age, sex, diet, environment, physiological conditions, and activity level. The hematology and blood chemistry of various Antillean manatee populations have been documented for Guyana [7], Mexico [8], Belize [9, 10], Brazil [11–14], and for the Florida subspecies (*T. manatus latirostris*) [6, 15–25]. However, this has not been ascertained for Antillean manatees inhabiting the Greater Antilles. Therefore, we sampled the population that inhabits the coastal waters of Puerto Rico, seeking to establish baseline complete blood cell count and serum chemistry reference intervals for this population of Antillean manatees.

## 2. Materials and Methods

### 2.1. Ethical Statement

The research has complied with all the relevant national regulations and institutional policies for the care and use of animals (Inter American University's Institutional Animal Care and Use Committee (IACUC) #6Mar2018).

### 2.2. Sample Collection

**Figure 1:**
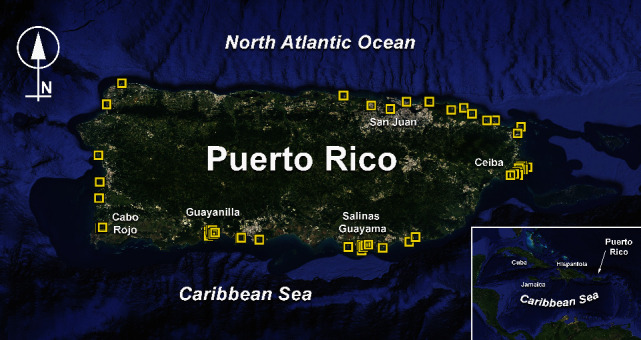
Capture or rescue locations for Antillean manatees included in this study.

We collected blood samples from 70 wild captured or rescued manatees from Puerto Rico from 1992 to 2020 (Table 1, Figure 1). Of these, 44 manatees were captured for health assessment and radiotelemetry studies, and 26 were primarily rescued as orphaned calves. Manatees were caught in nets deployed either along the shore or from a specialized net boat in open water [26] or rescued by hand in the case of orphaned calves. The captured manatees were immediately transported to a shaded area where they were kept moist at all times with the use of a water mister, buckets, and wet towels throughout the health assessment or brought into the Caribbean Manatee Conservation Center for veterinary examination. Data collected included sex, complete body measurements, tissue samples for genetic analysis, and blood draw. In addition, an experienced manatee veterinarian and veterinary technician, with the aid of marine biologists, conducted a complete physical examination including, among many others: visual external and behavioral assessment, heart rate, and respiratory rate [27] and other vital signs [28]. All manatees were considered healthy at the time of sampling. Manatees were categorized into 3 age classes based on total length and ear bone growth layer groups (GLGs) or known ages: calves (<175 cm, <2 years old), subadults (176–225 cm, 2–7 years old), and adults (>225 cm, >7 years old) [3]. Upon data and sample collection, the manatees were immediately returned to the water at their capture site, most fitted with a radio-transmitter for tracking studies [26], or in the case of orphaned calves, maintained in rehabilitation until completion of the time prescribed for their rehabilitation process and then released [29].

**Figure 2:**
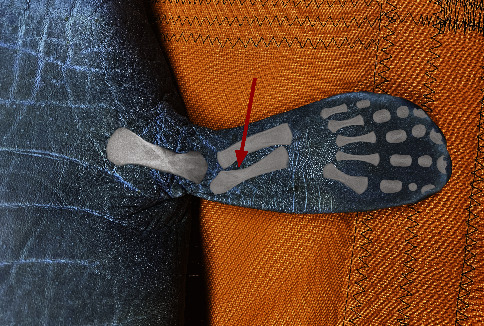
Location for venipuncture (red arrow) between the radius and ulna of the palmar section of the flipper of an Antillean manatee. Arm and hand bones overimposed graphically from an x-ray of the same individual.

**Figure 3:**
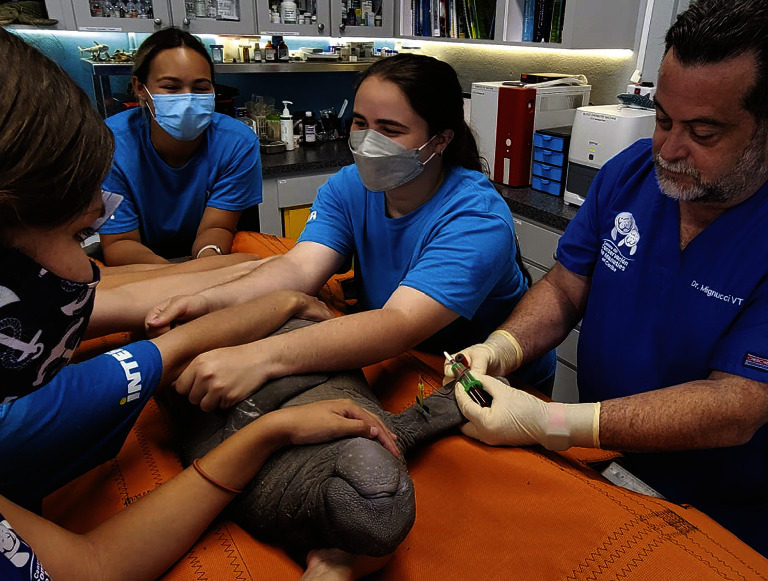
Venipuncture sampling on a calf Antillean manatee from Puerto Rico using a 21-gauge X 3/4-inch needle with a vacutainer and a “butterfly” set”.

**Figure 4:**
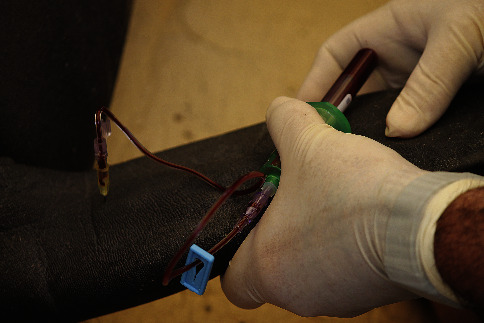
Venipuncture sampling on a subadult Antillean manatee from Puerto Rico using a 20-gauge X 1½-inch needle with a vacutainer and a 14-inch extension set.

Blood samples were obtained by venipuncture from the medial interosseous space of the radius and ulna, which constitutes the brachial vascular bundle (Figure 2). Before sample collection, the pectoral flipper was surgically scrubbed with povidone-iodine and alcohol or post-2017 with chlorhexidine scrub, chlorhexidine solution, and isopropyl alcohol. An 18–21-gauge, ¾–1½-inch needle with an attached “butterfly” BD Vacutainer blood collection set (Becton, Dickinson and Company, Franklin Lakes, NJ 07417, USA) or 14-inch extension set (International WIN, Limited, Kenneth Square, PA 19348, USA) was used depending on the size of the manatee (Figures 3 and 4). Because of the unique structure of the vascular system in this area, it was difficult to assess the specific vessel site in the vascular bundle (venous or arterial); thus, the blood collected was either or a combination of venous or arterial blood. Blood was collected first for serum chemistry analysis directly into 6 ml red top sterile vacutainer tubes with a silicone-coated interior (Becton, Dickinson and Company, Franklin Lakes, NJ 07417, USA), allowed to clot in a cool and shaded area, and then separated by centrifugation. Blood was then collected for a complete blood cell count (CBC) analysis into 4 ml lavender top sterile vacutainer tubes, which contained EDTA (K_2_EDTA; Becton, Dickinson and Company, Franklin Lakes, NJ 07417, USA) as the anticoagulant, agitated gently and kept cool until analyzed. Both serum and whole blood were kept refrigerated and transported to the same clinical reference laboratory for processing within 24 hours.

### 2.3. Complete Blood Cell Count

**Figure 5:**
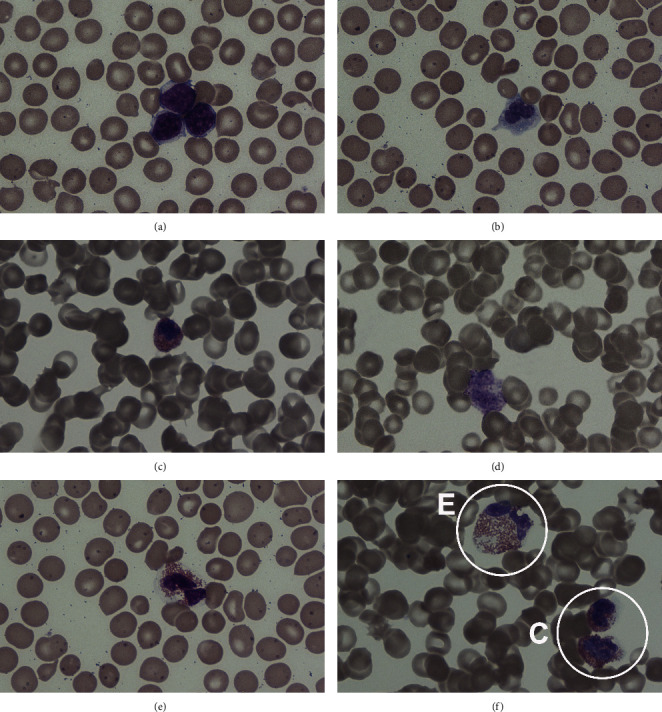
White blood cell types from an Antillean manatee from Puerto Rico: (a) lymphocytes, (b) monocyte, (c) eosinophil, (d) basophil, (e) heterophil, and (f) comparison between a heterophil (E) and two eosinophils (C). Wright-Giemsa, x100 objective.

Values for white blood cell count (WBC), red blood cell count (RBC), hemoglobin (HBG), hematocrit (HCT), platelet (PLTS), and the red blood indices of mean cell volume (MCV), mean cell hemoglobin (MCH), mean cell hemoglobin concentration (MCHC), and red cell distribution width (RDW) were obtained with a Cell-Dyn 3200 System Automated Hematology Analyzer (Abbott, Abbott Park, IL 60064 USA). In addition, a manual leukocyte differential was conducted under the microscope to allow for the evaluation of different leukocyte cell types, identifying and enumerating lymphocytes (LYMP), monocytes (MONO), eosinophils (EOSI), basophils (BASO), and heterophils (HETE), in percentages values. In automated CBC machines, heterophils are usually wrongly categorized as eosinophils due to their similarity in granulation morphology (Figure 5). Thus, it was essential to run manual leukocyte counts and, in doing so, train medical technologists and veterinarians in manatee leukocyte identification for correct categorization and counting.

### 2.4. Serum Chemistry

We performed a comprehensive metabolic panel (CMP) on all the serum samples with a VITROS 5, 1FS Chemistry System Analyzer (Ortho-Clinical Diagnostics, Rochester, NY 14626 USA). The resulting chemistry analytes were grouped based on six physiological processes: (1) liver-associated enzymes and pigments (lactate dehydrogenase, LDH; total bilirubin, TOT BIL); (2) muscle-associated enzymes (alanine aminotransferase, ALT; aspartate aminotransferase, AST; alkaline phosphatase, ALP; creatine phosphokinase, CPK); (3) kidney-associated compounds and products (blood urea nitrogen, BUN; creatinine, CREA; blood urea nitrogen-creatinine ratio, BUN:CREA; uric acid, UA); (4) sugars, lipids, and pancreatic-associated enzymes (glucose, GLU; triglycerides, TRIG; cholesterol, CHOL; amylase, AMY); (5) proteins (total protein, TOT PROT; albumin, ALB; globulin, GLOB; albumin-globulin ratio, ALB:GLOB); and (6) electrolytes (sodium, Na; chloride, Cl^−^; potassium, K; phosphate, PO_4_, or sometimes referred to as phosphorus, P; calcium, Ca; enzymatic carbon dioxide, CO_2_; anion gap, AG).

### 2.5. Statistical Analysis

We used Microsoft Excel for Mac (Microsoft Corp. Version 12.2.8) for statistical analyses. Summary statistics (sample size, mean, maximum value, minimum value, and standard deviation) of hematology and serum chemistry values were calculated on all the samples for every parameter. Minimum and maximum intervals using ±2 standard deviation around the mean were calculated, and values outside this range were considered outliers and eliminated. Means and standard deviations were then recalculated, and new reference ranges were determined as ±1 standard deviation around the mean. An unpaired two-sample *T*-test was done comparing males versus females and adults versus calves to see if there were any significant differences (*p* ≤ 0.05) between these. Subadults were not included in the latter due to small sample size.

## 3. Results

### 3.1. Sample Description

Seventy captured or rescued manatees from Puerto Rico were included in the study (Table 1). Of these, 33 were males, and 37 were females; 23 were calves, 6 were subadults, and 41 were adult individuals. Sex by age group was evenly distributed, with 9 male and 14 female calves, 3 male and 3 female subadults, and 21 males and 20 female adults. The distribution of manatees sampled around the island was also evenly distributed (Figure 1). However, directed telemetry and health assessment captures were conducted in Ceiba, Salinas, Guayama (Jobos Bay), Guayanilla, Cabo Rojo, and Mayagüez.

### 3.2. Complete Blood Cell Count

Hematological parameters were obtained for the entire population sampled, separately for calves, subadults, adults (Table 2), males, and females (Table 3). Leukocytes in the Puerto Rico manatee population were composed primarily of heterophils (52.3%, range of 38–67%) and lymphocytes (42.8%, range of 28–57%), with few monocytes, and rare eosinophils and basophils. We found significant differences between calf and adult manatees in red blood cell count, hemoglobin, hematocrit, mean cell volume, white blood cell count, and basophils. All were found to be higher in calves, except for mean cell volume and basophils, which were lower than in adults (Table 2). Manatee neonates and calves had noticeably increased red blood cell count (3.0–3.8 10^6^/mm^3^), decreasing after 2–3 months of age (data not shown). White blood cell count was also found to be higher in calves, leveling to normal values after 3 months of age (data not shown). There were no observed significant differences between males and females regarding hematology values (Table 3), except for red cell distribution width (*p*=0.007) and platelets (*p*=0.047), which were slightly higher in females.

### 3.3. Serum Chemistry

Serum chemistry parameters were also obtained for the entire population sampled and separately for calves, subadults, adults (Table 4), males, and females (Table 5). Significant differences (*p* ≤ 0.05) were found in all chemistry analytes when comparing adults versus calves, except aspartate aminotransferase, alkaline phosphatase, creatine phosphokinase, lactic dehydrogenase, blood urea nitrogen, blood urea nitrogen-creatinine ratio, triglycerides, cholesterol, amylase, globulin, and potassium (Table 4). Adult manatees had a higher mean value for alanine aminotransferase, creatinine, uric acid, glucose, total protein, albumin, albumin-globulin ratio, sodium, chloride, potassium, and anion gap. Calves had a lower mean value for total bilirubin, calcium, and enzymatic carbon dioxide. When comparing males versus females, no significant differences were found in all chemistry analytes (Table 5).

## 4. Discussion

We establish the reference intervals of hematology and blood chemistry for the population of Antillean manatees in Puerto Rico and compare them with those established for manatees from Belize, Brazil, Florida, Guyana, and Mexico. Blood reference intervals can differ among populations of the same species and vary according to age, sex, physiological condition, degree of physical activity, and environment [30]. They can also be altered by collecting and testing methods. They may also vary within individuals of the same population, suggesting the clinical need for veterinarians to compare and contrast hematology and blood chemistry results on a particular manatee patient as it develops medically during treatment and in long-term care. While differences in immunoglobulin G (IgG) reference values for manatees from Florida, Colombia, and Puerto Rico were found [31] and comparisons were made on hematological and serum chemistry reference ranges between Antillean manatees in Guyana and Florida manatees [7], these studies found that the majority of hematological and serum chemistry results were similar to those reported for the Florida manatee. Blood parameters of Antillean manatees in Brazil were also evaluated, and differences between sexes and environments were discussed [14]. Tabulated reference ranges for both subspecies of West Indian manatees (Antillean and Floridian) from Guyana, Mexico, Belize, Brazil, and Florida (Table 6 and 7) were used to discuss similarities or differences between these populations in comparison to hematology and blood chemistry reference ranges found for Puerto Rico.

### 4.1. Complete Blood Cell Count

Erythrocyte and total leukocyte counts measured in this study were similar to those previously reported for manatees in Guyana, Mexico, Belize, Brazil, and Florida [6–9, 14, 25] (Table 6). Red blood cell count was highest in neonates (>3.0 × 10^6^/mm^3^) and decreased as manatees grew and learned to dive [6, 32], as evident in significant variation among age classes observed in the study (Table 2). This is opposite to erythrocyte count in common bottlenose dolphins (*Tursiops truncatus*), pinnipeds, and terrestrial mammals as they mature in age [33].

Leukocyte counts in manatees tend to be slightly lower than in most domestic mammals but in general similar to several whale and dolphin species [6, 25]. For example, the range for Antillean manatees in Puerto Rico reported here is between 4.0 and 8.0 × 10^9^/L, with calves having a slightly higher reference range (5.3 and 9.3 × 10^9^/L) (Table 2). However, it is advised that a manatee with a white blood cell count above 8.0 × 10^9^/L should be closely monitored, and anything above 10 × 10^9^/L be considered abnormal. It is of extreme importance that white blood cell types be ascertained using a manual method, rather than by machine, and the technician reading the slide be forewarned, as heterophils may be mistaken for eosinophils given their granulocyte nature (Figure 5). Eosinophils in manatees are rarely found, usually 0–4%. Eosinophilia reported for manatees was usually performed by untrained laboratory technicians or veterinarians, which mistakenly read granular heterophils as eosinophils (AAMG personal observation).

### 4.2. Serum Chemistry

#### 4.2.1. Liver-Associated Enzymes and Pigments

Wide ranges of lactic dehydrogenase observed in our marine-dwelling manatees as well as in other marine mammals may be due to muscle exertion associated with diving. Although manatees in Puerto Rico are not usually deep or long divers (<10 m, <5–10 min), differences in reported lactic dehydrogenase ranges may be due also to the administration of intramuscular injections and the manipulation prior to or during sampling [34]. Artificial changes in lactic dehydrogenase levels were reported in cetaceans due to severe hemolysis similar to those observed in individuals that received intramuscular injections but of a lesser magnitude [35]. Total bilirubin values in Puerto Rico were significantly lower in adults than in calf manatees, as calves have a higher metabolic demand for protein absorption from their mother's milk and complementary vegetable diet (Table 4). However, overall total bilirubin values were similar to those found in other studies in Guyana, Mexico, Belize, Brazil, and Florida [6–9, 14, 24] (Table 7).

#### 4.2.2. Muscle-Associated Enzymes

While Bossart et al. [6] included alanine aminotransferase, aspartate aminotransferase, and alkaline phosphatase in the liver-associated enzymes, to date, these analytes are considered more indicative of the muscular system [22]. Alanine aminotransferase values for Puerto Rico were significantly higher in adults compared to calves (Table 4) and were similar to Mexico, Belize, and Florida [6, 8, 9, 24] but higher than those found in Brazil [14]. Aspartate aminotransferase appears to be of little or no clinical value in manatees [6]. However, values for Puerto Rico were comparable to those described for Florida [6, 24], Guyana [7], and Brazil [14] but lower than in Mexico and Belize [8, 9]. Increased alkaline phosphatase may be present in growing, young mammals in some terrestrial species associated with osteoblastic activity [36], as seen in our reference ranges of Puerto Rican manatee calves versus adults. Although no detailed studies have been conducted in marine mammals, similar trends for alkaline phosphatase activity may also exist [6], and decreased alkaline phosphatase values appear to indicate older individuals, pernicious anemia, hypothyroidism, inanition, or decreased osteoblastic activity [6]. In manatees from Puerto Rico, alkaline phosphatase values for calves were clearly higher than in subadult and adult manatees.

Creatine phosphokinase values for Puerto Rico were similar to those found for Guyana [7], Mexico [8], and Florida [6]. However, they were notably different from those reported by Florida's later study [24]. Creatine phosphokinase is an important parameter to measure in manatees in rehabilitation, as it is indicative of handling stress [6] and intestinal tissue remodeling due to colitis and enteritis. In our experience, in digestively compromised Antillean manatees, creatine phosphokinase values rapidly surpass the 500–2,000 U/L levels, and intestinal inflammation is not resolved until the values return to normal ranges (68–132 U/L). Therefore, rapid multifaceted treatment is warranted in these cases, or the chances of the manatee's demise increase as time passes from necrotizing enteritis and pneumatosis intestinalis.

#### 4.2.3. Kidney-Associated Compounds and Products

Reference intervals for blood urea nitrogen for Puerto Rico manatees were slightly lower than those initially established for the Florida manatee [6] but similar to those in Guyana [7], Mexico [8], Belize [9], and Florida's later study [24]. Blood urea nitrogen reacts to a complex combination of several variables such as nutritional, age, and metabolic and physiologic conditions during sampling and restraint. Decreased blood urea nitrogen may reflect their herbivorous diet [34]. Although Florida and the Antillean manatee diet are similar, the difference in blood urea nitrogen ranges may reflect different feeding behaviors during the changing seasons in Florida. Starvation and liver failure cause blood urea nitrogen levels to decrease [6].

Creatinine reference ranges in Puerto Rico were significantly different between calves and adults, being higher in the latter (Table 4). However, creatinine values were similar to those established for Antillean manatees in Guyana [7], Belize [9], Brazil [14], and Florida [6], but not when compared to later study of Mexico [8] or Florida [24]. Variations associated with food intake, type of food, and salinity of environment in Florida manatees were suggested to possibly be affecting serum creatinine levels [20]. Rehabilitated Antillean manatees from Puerto Rico exhibited an increased serum creatinine following release back to the wild; while blood urea nitrogen and other blood parameters remained within their normal baseline ranges. An increase in creatinine was documented on all rehabilitated and released Antillean manatees in Puerto Rico immediately after being transferred to a sea pen during a soft release back into the wild. After the manatees acclimated to the new environment and learned to find a freshwater source, creatinine levels decreased in subsequent veterinary examinations.

Uric acid parameters were significantly higher in manatee calves from Puerto Rico when compared to adult manatees (Table 4), most probably due to the higher protein content in the calves' diet. However, uric acid values were not available from Guyana, Mexico, Belize, and Florida to further understand any differences between populations.

#### 4.2.4. Sugars, Lipids, and Pancreatic-Associated Enzymes

Adult manatees in Puerto Rico showed significantly higher values for glucose in comparison to calves (Table 4). Hypoglycemia in rescued calves or debilitated manatees is a significant problem that must be addressed urgently upon admittance to a critical care facility. Glucose and triglyceride levels were similar among Puerto Rico, Guyana, Mexico, Belize, Brazil, and Florida populations. However, cholesterol levels from all Antillean manatees throughout the countries were similar but appreciably lower than those found in Florida manatees [6, 24], given that Florida manateesare larger in size and have higher fat reserves from being a northern and subtropical subspecies.

#### 4.2.5. Proteins

Values for total protein, albumin, and albumin-globulin ratio were significantly higher in adult manatees in Puerto Rico than in calf manatees (Table 4). Plasma or serum proteins move between the blood and other fluids. The total concentration of all proteins in the blood may vary depending on changes in the volume of water or the amount of the individuals' proteins. Reference intervals for total protein established for the Puerto Rico population were slightly lower than those reported for Mexico [8] and Florida [6, 24] but similar to those in Guyana, Belize, and Brazil [7, 9, 14]. An increase in total proteins, indicative of dehydration, was observed, while manatees were in the more severe diet reduction phase of a simulated release [20]. Variations between freshwater and marine diets may explain the difference in total protein values between captive calves and free-ranging adults. Additionally, blood sample quality should be considered when evaluating total protein values due to an artificial increase caused by lipemia, icterus, and hemolysis. Albumin serves as a protein carrier, and given that it is typically higher in most marine mammals, the use of automated analyzers using human standards may give erroneous results [37]. As an early indicator of hepatic disease in marine mammals, the use of albumin is limited since it appears that these have a tremendous reserve capacity for hepatic albumin production [6]. Albumin reference intervals for manatees in Puerto Rico were slightly lower than those reported for both Antillean and Florida manatees in other compared countries. These values were confirmed by protein electrophoresis, the preferred and recommended detection method [6]. Elevated albumin levels occur with dehydration and shock, while malnutrition and gastrointestinal disease can decrease the levels among other circumstances. The small variation in albumin levels between captive calves and free-ranging adults was probably an effect of some or all of these factors. Increased albumin due to dehydration may be caused by the limited availability of freshwater sources for free-ranging adults, in recently released subadults in the process of acclimating to the saline environment, and in rescued calves that are typically brought in with colic and other gastrointestinal complications due to malnourishment.

#### 4.2.6. Electrolytes

Electrolytes analytes were significantly different between adults and calves, except for phosphate. Sodium, chloride, potassium, and anion gap were higher in adults, while calcium and enzymatic carbon dioxide were higher in calves. However, electrolyte values across countries of the Antillean subspecies and the Florida subspecies were similar.

## 5. Conclusions

Here, we establish the normal reference intervals for the population of Antillean manatees in the waters around the island of Puerto Rico (Tables 6 and 7). Most findings in this study were similar to those previously reported for other West Indian manatee populations of both subspecies from Guyana, Mexico, Belize, Brazil, and Florida. Factors to be considered when evaluating and explaining these similarities or differences include diet, time from last feeding, water composition (salinity, temperature, pH, etc.), body condition, capture stress, and health status. This study's interpretation of hematology and blood chemistry data was complicated since most adult samples were collected from targeted free-ranging manatees for radiotelemetry studies, and most calves were in a captive environment for medium-term care after rescue and health stabilization. Therefore, blood reference ranges provided herein should be considered complementary guidelines for veterinary examinations and health assessments. If possible, baseline blood parameters should be established for each individual manatee as a patient or during long-term care before declaring any value as truly abnormal.

## Figures and Tables

**Table 1:** Antillean manatees sampled in Puerto Rico for this study, including those free-ranging and those rescued and in rehabilitation between 1991 and 2020.

Date	Field number	Name	Sex	Length (cm)	Weight (kg)	Age class	Locality
4 Dec 1991	NEPST175	Moisés	M	115	28.6	C	Toa Baja
13 Apr 1992	CPR9201	CPR9201	F	251	—	A	Ceiba
16 Apr 1992	TPR01	Taino	M	276	360	A	Ceiba
16 Apr 1992	TPR02	Caribe	M	276	386	A	Ceiba
18 Apr 1992	CPR9202	CPR9202	M	242	—	A	Ceiba
18 Apr 1992	CPR9203	CPR9203	M	294	—	A	Ceiba
18 Apr 1992	TPR03	Ceiba	M	275	—	A	Ceiba
19 May 1993	TPR04	Simu	M	244	—	A	Ceiba
20 May 1993	TPR05	Mac	M	264	—	A	Ceiba
22 May 1993	CPR9301	CPR9301	M	230	—	A	Ceiba
22 May 1993	CPR9302	CPR9302	F	210	—	SA	Ceiba
22 May 1993	TPR06	Eddie T.	M	273	—	A	Ceiba
23 May 1993	NEPST212	Glauko	M	122	31	C	San juan
7 Sep 1994	TPR08	Judini	M	276	—	A	Ceiba
21 Jul 1995	NEPST380	Marina	F	102	17	C	Guayama
28 Aug 1996	NEPST519	Katsi	F	118	30	C	Cabo rojo
7 Aug 1997	CPR9701	Bea	F	125	—	C	Mayagüez
7 Aug 1997	TPR09	Guanajibo	M	241	—	A	Mayagüez
7 Aug 1997	TPR10	Sara	F	264	—	A	Mayagüez
11 Jun 1999	NEPST548	Nina	F	131	40	C	Guayama
23 Jul 1999	NEPST556	Yuisa	F	95	16	C	Loíza
14 Sep 1999	NEPST574	Conquistador	M	108	23	C	Fajardo
11 Nov 2002	NEPST852	Santa Cruz	F	238	195	A	Ponce
3 Jun 2003	NEPST861	Anani	F	308	440	A	Arroyo
17 Jul 2003	TPR13	Albanai	F	299	—	A	Cabo rojo
18 Jul 2003	TPR14	Guami	M	250	270	A	Cabo rojo
20 Jul 2003	CPR0301	Iro	M	212	161	SA	Guayanilla
20 Jul 2003	TPR15	Atabey	F	296	416	A	Guayanilla
21 Jul 2003	CPR0302	CPR0302	F	232	254	A	Guayanilla
23 Jul 2003	NEPST592	Rafael	M	247	289	A	Luquillo
26 Jul 2003	NEPST868	Camilia	F	99	17	C	Isabela
3 Nov 2003	TPR11	Joyuda	F	296	—	A	Cabo rojo
4 Nov 2003	TPR16	Igor	M	287	—	A	Cabo rojo
5 Nov 2003	TPR17	Eco	M	310	—	A	Guayanilla
5 Nov 2003	TPR18	Electra	F	267	—	A	Guayanilla
6 Nov 2003	TPR19	Esoubi	M	249	—	A	Guayanilla
7 Nov 2003	CPR0303	Guanina	F	236	—	A	Guayanilla
7 Jun 2004	TPR20	Gazelle	F	288	401	A	Guayanilla
7 Jun 2004	TPR21	Sally	F	297	445	A	Guayanilla
8 Jun 2004	TPR22	Beethoven	M	256	310	A	Guayanilla
10 Jun 2004	CPR0401	CPR0401	F	193	—	SA	Cabo rojo
10 Jun 2004	TPR23	Coral	F	261	—	A	Cabo rojo
23 Aug 2004	NEPST892	Iani	F	111	26	C	Ponce
22 Nov 2004	NEPST895	Siani	F	109	23	C	Aguadilla
18 Mar 2005	NEPST899	Guaili	F	115	29	C	Guayama
25 Apr 2005	TPR25	Maritzilla	F	296	—	A	Ceiba
26 Apr 2005	TPR26	Rosa	F	303	—	A	Ceiba
27 Apr 2005	CPR0501	CPR0501	M	222	—	SA	Ceiba
28 Apr 2005	TPR27	India	F	255	—	A	Ceiba
29 Apr 2005	TPR28	Monty	M	273	300	A	Ceiba
29 Apr 2005	TPR29	Marietta	F	264	—	A	Ceiba
29 Apr 2005	TPR30	PJ	M	225	—	SA	Ceiba
30 Apr 2005	TPR31	Marina	F	270	—	A	Ceiba
1 May 2005	TPR32	Flipa	F	250	—	A	Ceiba
2 May 2005	TPR33	Treso	M	252	—	A	Ceiba
30 Sep 2005	NEPST910	El Tuque	M	111	29	C	Ponce
18 Sep 2006	NEPST927	Guarionex	M	104	17	C	Fajardo
18 May 2011	NEPST940	Aramana	M	107	20	C	Dorado
15 May 2013	NEPST948	Mayagua	F	138	40	C	Mayagüez
21 Jul 2013	NEPST951	Yuisa	F	129	33	C	Loíza
27 Oct 2014	NEPST958	Río Grande	M	222	227	A	Río Grande
3 Aug 2015	CCMPR150803Tm01	Tureygua	M	116	26	C	Isabela
7 Jun 2016	CCMPR160607Tm01	Mabo	M	124	31	C	San juan
10 Aug 2017	CPR1701	Abey	F	274	297	A	Salinas
10 Aug 2017	CPR1702	Baracutey	M	229	211	A	Guayama
11 Aug 2017	CPR1703	Biminí	F	195	119	SA	Guayama
3 Sep 2019	CCM190903Tm01	Guamaní	F	118	26	C	Salinas
30 Jan 2020	CCM191227Tm01	Loíza	F	114	25	C	Loíza
13 Jul 2020	CCM200524Tm01	Taicaraya	F	137	46	C	Luquillo
27 Jul 2020	CCM200705Tm01	Bajarí	M	121	29	C	Arroyo

*Note.* M = male, F = female, A = adult, SA = subadult, and C = calf.

**Table 2:** Mean and standard deviation hematology values for Antillean manatees from Puerto Rico for all age classes (calves, subadults, and adults; *n* = 70): calves only, *n* = 23; subadults only, *n* = 6; and adults only, *n* = 41, with ±1 standard deviation ranges in parentheses. Significant differences using *p* values between calves and adults are indicated with an asterisk (^*∗*^).

Parameter	All manatee samples		Calves		Subadults		Adults		*p* value
	Mean ± SD	Range	Mean ± SD	Range	Mean ± SD	Range	Mean ± SD	Range	Calves vs. adults
WBC (10^9^/L)	6.0 ± 2.0	(4.0–8.0)	7.3 ± 2.0	(5.3–9.3)	4.9 ± 1.8	(3.1–6.7)	5.4 ± 1.7	(3.8–7.1)	0.004^*∗*^
RBC (10^6^/mm^3^)	2.7 ± 0.6	(2.1–3.3)	3.4 ± 0.4	(3.0–3.8)	2.3 ± 0.1	(2.2–2.4)	2.3 ± 0.02	(2.1–2.6)	≤0.001^*∗*^
HBG (g/dL)	11.2 ± 2.3	(9.0–14)	13.8 ± 1.5	(12–15)	9.4 ± 0.5	(8.9–9.8)	9.8 ± 0.8	(8.9–11)	≤0.001^*∗*^
HCT (%)	34.6 ± 6.4	(28–41)	41.6 ± 4.1	(38–46)	30.3 ± 4.0	(26–34)	30.7 ± 3.5	(27–34)	≤0.001^*∗*^
PLTS (10^3^/mm^3^)	298.3 ± 91.3	(207–390)	306.7 ± 112.9	(194–420)	390.0 ± 79.4	(311–469)	283.8 ± 80.0	(204–364)	0.563
Red blood cell indices									
MCV (fL)	128.6 ± 8.2	(120–137)	124.5 ± 7.2	(117–132)	124.9 ± 8.1	(117–133)	131.7 ± 7.7	(124–140)	0.001^*∗*^
MCH (pg)	41.1 ± 1.4	(40–43)	40.9 ± 1.7	(39–43)	40.6 ± 0.7	(40–41)	41.3 ± 1.2	(40–43)	0.424
MCHC (g/dL)	32.2 ± 1.2	(31–34)	32.5 ± 1.2	(31–34)	32.5 ± 1.5	(31–34)	32.0 ± 1.3	(31–33)	0.124
RDW (%)	18.0 ± 2.8	(15–21)	17.9 ± 2.8	(15–21)	17.2 ± 2.1	(15–19)	18.2 ± 2.9	(15–21)	0.681
White blood cell differential									
LYMP (%)	42.8 ± 14.5	(28–57)	38.2 ± 16.5	(22–55)	54.0 ± 8.0	(46–62)	44.2 ± 13.0	(31–57)	0.165
MONO (%)	3.6 ± 2.3	(1–6)	4.1 ± 2.7	(1–7)	2.5 ± 1.7	(1–4)	3.5 ± 2.2	(1–6)	0.414
EOSI (%)	1.0 ± 1.4	(0–3)	0.6 ± 1.1	(0–2)	2.3 ± 2.1	(0–4)	1.1 ± 1.5	(0–3)	0.118
BASO (%)	0.3 ± 0.5	(0–1)	0.0 ± 0.2	(0–0)	0.5 ± 0.6	(0–1)	0.5 ± 0.6	(0–1)	≤0.001^*∗*^
HETE (%)	52.3 ± 14.7	(38–67)	57.1 ± 16.9	(40–74)	40.8 ± 7.5	(33–48)	50.7 ± 13.0	(38–64)	0.145

*Note.* WBC = white blood cell count, RBC = red blood cell count, HBG = hemoglobin, HCT = hematocrit, PLTS = platelet count, MCV = mean corpuscular volume, MCH = mean corpuscular hemoglobin, MCHC = mean corpuscular hemoglobin concentration, RDW = red cell distribution width, LYMP = lymphocytes, MONO = monocytes, EOSI = eosinophils, BASO = basophils, and HETE = heterophils.

**Table 3:** Mean and standard deviation hematology values for Antillean manatees from Puerto Rico for all sex classes (males and females, *n* = 70): males only, *n* = 33, and females only, *n* = 37, with ±1 standard deviation ranges in parentheses. Significant differences using *p* values are indicated with an asterisk (^*∗*^).

Parameter	All manatee samples		Males		Females		*p* values
	Mean ± SD	Range	Mean ± SD	Range	Mean ± SD	Range	
WBC (10^9^/L)	6.0 ± 2.0	(4.0–8.0)	5.8 ± 1.9	(3.9–7.7)	6.2 ± 2.1	(4.1–8.2)	0.539
RBC (10^6^/mm^3^)	2.7 ± 0.6	(2.1–3.3)	2.6 ± 0.6	(2.1–3.2)	2.7 ± 0.6	(2.1–3.4)	0.522
HBG (g/dL)	11.2 ± 2.3	(9.0–14)	10.9 ± 2.0	(8.9–13)	11.5 ± 2.4	(9.0–14)	0.322
HCT (%)	34.6 ± 6.4	(28–41)	34.2 ± 6.1	(28–40)	34.9 ± 6.8	(28–42)	0.646
PLTS (10^3^/mm^3^)	298.3 ± 91.3	(207–390)	269.9 ± 81.9	(188–352)	323.2 ± 93.3	(230–417)	0.047^*∗*^
Red blood cell indices							
MCV (fL)	128.6 ± 8.2	(120–137)	128.3 ± 7.9	(120–136)	128.8 ± 8.6	(120–137)	0.824
MCH (pg)	41.1 ± 1.4	(40–43)	40.8 ± 1.3	(40–42)	41.4 ± 1.4	(40–43)	0.168
MCHC (g/dL)	32.2 ± 1.2	(31–34)	32.2 ± 1.1	(31–33)	32.2 ± 1.4	(31–34)	0.941
RDW (%)	18.0 ± 2.8	(15–21)	16.9 ± 2.2	(15–19)	18.9 ± 2.9	(16–22)	0.007^*∗*^
White blood cell differential							
LYMP (%)	42.8 ± 14.5	(28–57)	42.2 ± 10.5	(32–53)	43.2 ± 17.2	(26–60)	0.776
MONO (%)	3.6 ± 2.3	(1–6)	3.4 ± 2.3	(1–6)	3.8 ± 2.4	(1–6)	0.584
EOSI (%)	1.0 ± 1.4	(0–3)	1.1 ± 1.5	(0–3)	0.9 ± 1.5	(0–2)	0.618
BASO (%)	0.3 ± 0.5	(0–1)	0.4 ± 0.6	(0–1)	0.3 ± 0.5	(0–1)	0.304
HETE (%)	52.3 ± 14.7	(38–67)	52.8 ± 10.7	(42–64)	51.8 ± 17.4	(34–69)	0.783

*Note.* WBC = white blood cell count, RBC = red blood cell count, HBG = hemoglobin, HCT = hematocrit, PLTS = platelet count, MCV = mean corpuscular volume, MCH = mean corpuscular hemoglobin, MCHC = mean corpuscular hemoglobin concentration, RDW = red cell distribution width, LYMP = lymphocytes, MONO = monocytes, EOSI = eosinophils, BASO = basophils, and HETE = heterophils.

**Table 4:** Mean serum chemistry values for manatees from Puerto Rico for all samples (calves, subadults, and adults, *n* = 70): calves only, *n* = 23; subadults only, *n* = 6; and adults only, *n* = 41, with ±1 standard deviation range in parentheses. Abbreviations for parameters are detailed in the Materials and Methods section. Significant differences using *p* values between calves and adults are indicated with an asterisk (^*∗*^).

Parameter	All manatee samples		Calves		Subadults		Adults		*p* value
	Mean ± SD	Range	Mean ± SD	Range	Mean ± SD	Range	Mean ± SD	Range	Calves vs. Adults
Liver-associated enzymes and pigments									
LDH (U/L)	425.3 ± 164.6	(261–590)	394.6 ± 174.1	(220–569)	538.2 ± 144.6	(394–683)	420.2 ± 159.7	(261–580)	0.620
TOT BILI (mg/dL)	0.2 ± 0.1	(0.1–0.3)	0.3 ± 0.2	(0.1–0.4)	0.1 ± 0.04	(0.1–0.2)	0.2 ± 0.1	(0.1–0.3)	0.009^*∗*^
Muscle-associated enzymes									
ALT (U/L)	15.4 ± 7.1	(8.3–23)	12.0 ± 6.5	(5.5–18)	18.5 ± 5.5	(13–24)	16.8 ± 7.1	(9.7–24)	0.011^*∗*^
AST (U/L)	11.4 ± 5.2	(6.1–17)	10.3 ± 4.9	(5.4–15)	13.5 ± 6.4	(7.1–20)	11.9 ± 5.5	(6.4–17)	0.316
ALP (U/L)	78.2 ± 20.4	(58–99)	86.9 ± 20.6	(66–108)	70.2 ± 20.7	(49–91)	76.2 ± 19.8	(56–96)	0.096
CPK (U/L)	99.8 ± 32.3	(68–132)	85.8 ± 24.1	(62–110)	119.4 ± 20.9	(99–140)	101.4 ± 34.9	(67–136)	0.136
Kidney-associated compounds and products									
BUN (mg/dL)	4.2 ± 2.1	(2.1–6.3)	4.1 ± 2.5	(1.6–6.5)	5.2 ± 2.2	(3.0–7.4)	4.1 ± 2.0	(2.2–6.1)	0.896
CREA (mg/dL)	1.5 ± 0.4	(1.1–1.9)	1.3 ± 0.4	(0.9–1.8)	1.5 ± 0.5	(1.0–2.1)	1.6 ± 0.4	(1.3–2.0)	0.014^*∗*^
BUN:CREA	2.1 ± 0.7	(1.4–2.8)	1.7 ± 0.8	(0.9–2.4)	2.3 ± 0.4	(1.9–2.7)	2.2 ± 0.7	(1.5–2.9)	0.078
UA (mg/dL)	0.9 ± 0.5	(0.4–1.4)	1.2 ± 0.4	(0.8–1.6)	0.8 ± 0.6	(0.2–1.4)	0.8 ± 0.5	(0.3–1.3)	0.009^*∗*^
Sugars, lipids, and pancreatic-associated enzymes									
GLU (mg/dL)	91.2 ± 18.5	(73–110)	78.5 ± 14.6	(64–93)	86.7 ± 21.5	(65–108)	97.0 ± 16.9	(80–114)	0.001^*∗*^
TRIG (mg/dL)	109.3 ± 25.2	(84–135)	110.7 ± 28.0	(83–139)	88.4 ± 17.5	(71–106)	112.4 ± 24.4	(88–137)	0.867
CHOL (mg/dL)	117.5 ± 25.7	(92–143)	138.0 ± 14.9	(123–153)	115 ± 19.8	(95–135)	116.2 ± 26.7	(90–143)	0.105
AMY (U/L)	599.7 ± 149.1	(451–749)	667.0 ± 179.3	(488–846)	—	—	573.5 ± 145.3	(428–719)	0.399
Proteins									
TOT PROT (g/dL)	7.0 ± 0.5	(6.4–7.5)	6.7 ± 0.6	(6.1–7.3)	6.8 ± 0.4	(6.3–7.2)	7.1 ± 0.4	(6.7–7.6)	0.004^*∗*^
ALB (g/dL)	3.9 ± 0.5	(3.5–4.4)	3.7 ± 0.5	(3.2–4.3)	3.9 ± 0.4	(3.5–4.4)	4.1 ± 0.4	(3.7–4.5)	0.012^*∗*^
GLOB (g/dL)	2.9 ± 0.4	(2.5–3.3)	3.0 ± 0.4	(2.6–3.5)	2.8 ± 0.5	(2.3–3.3)	2.8 ± 0.4	(2.4–3.1)	0.143
ALB:GLOB	1.4 ± 0.3	(1.1–1.7)	1.2 ± 0.3	(0.9–1.5)	1.5 ± 0.3	(1.1–1.8)	1.4 ± 0.2	(1.2–1.7)	0.032^*∗*^
Electrolytes									
Na (mmol/L)	151.0 ± 5.5	(146–157)	147.8 ± 5.1	(143–153)	151.0 ± 6.2	(145–157)	152.6 ± 5.0	(148–158)	≤0.001^*∗*^
Cl^−^ (mmol/L)	99.6 ± 6.3	(93–106)	97.3 ± 6.5	(91–104)	99.9 ± 6.1	(94–106)	101.0 ± 5.9	(95–107)	0.036^*∗*^
K (mmol/L)	5.3 ± 0.7	(4.6–5.9)	4.8 ± 0.5	(4.3–5.2)	5.5 ± 0.5	(5.0–5.9)	5.5 ± 0.6	(4.9–6.1)	≤0.001^*∗*^
PO_4_ (mg/dL)	5.9 ± 1.1	(4.8–7.0)	6.2 ± 1.1	(5.1–7.3)	6.3 ± 1.3	(5.0–7.5)	5.7 ± 1.1	(4.5–6.8)	0.110
Ca (mg/dL)	9.8 ± 0.7	(9.2–11)	10.1 ± 0.6	(9.6–11)	9.5 ± 0.5	(9.0–10)	9.7 ± 0.7	(9.0–10)	0.020^*∗*^
CO_2_ (mmol/L)	26.7 ± 9.5	(17–36)	33.7 ± 4.5	(29–38)	23.9 ± 11.7	(12–36)	23.0 ± 9.3	(14–32)	≤0.001^*∗*^
AG (mmol/L)	27.9 ± 10.6	(17–39)	19.9 ± 6.5	(13–26)	32.4 ± 11.7	(21–44)	32.2 ± 9.7	(23–42)	≤0.001^*∗*^

**Table 5:** Mean and standard deviation hematology values for Antillean manatees from Puerto Rico for all sex classes (males and females, *n* = 70): males only, *n* = 33, and females only, *n* = 37, with ±1 standard deviation ranges in parentheses. Significant differences using *p* values are indicated with an asterisk (^*∗*^).

Parameter	All manatee samples		Males		Females		*p* values
	Mean ± SD	Range	Mean ± SD	Range	Mean ± SD	Range	
Liver-associated enzymes and pigments							
LDH (U/L)	425.3 ± 164.6	(261–590)	392.3 ± 155.0	(237–547)	458.4 ± 170.0	(288–628)	0.127
TOT BILI (mg/dL)	0.2 ± 0.1	(0.1–0.3)	0.2 ± 0.1	(0.1–0.3)	0.2 ± 0.1	(0.1–0.3)	0.573
Muscle-associated enzymes							
ALT (U/L)	15.4 ± 7.1	(8.3–23)	14.6 ± 8.1	(6.5–23)	16.0 ± 6.3	(9.8–22)	0.435
AST (U/L)	11.4 ± 5.2	(6.1–17)	9.9 ± 4.9	(5.0–15)	12.8 ± 5.3	(7.5–18)	0.053
ALP (U/L)	78.2 ± 20.4	(58–99)	75.1 ± 20.3	(55–95)	81.0 ± 20.4	(61–101)	0.271
CPK (U/L)	99.8 ± 32.3	(68–132)	101.2 ± 32.1	(69–103)	98.5 ± 33.2	(65–132)	0.785
Kidney-associated compounds and products							
BUN (mg/dL)	4.2 ± 2.1	(2.1–6.3)	4.6 ± 2.2	(2.4–6.8)	3.9 ± 2.1	(1.9–6.0)	0.222
CREA (mg/dL)	1.5 ± 0.4	(1.1–1.9)	1.5 ± 0.4	(1.1–2.0)	1.5 ± 0.4	(1.1–1.9)	0.771
BUN:CREA	2.1 ± 0.7	(1.4–2.8)	2.3 ± 0.7	(1.5–3.0)	2.0 ± 0.7	(1.2–2.7)	0.234
UA (mg/dL)	0.9 ± 0.5	(0.4–1.4)	0.9 ± 0.5	(0.4–1.5)	0.9 ± 0.5	(0.4–1.4)	0.723
Sugars, lipids, and pancreatic-associated enzymes							
GLU (mg/dL)	91.2 ± 18.5	(73–110)	90.4 ± 18.7	(72–109)	91.8 ± 18.6	(73–110)	0.771
TRIG (mg/dL)	109.3 ± 25.2	(84–135)	109.8 ± 24.2	(86–134)	108.8 ± 26.6	(82–135)	0.900
CHOL (mg/dL)	117.5 ± 25.7	(92–143)	121.0 ± 23.5	(97–144)	114.3 ± 27.7	(87–142)	0.380
AMY (U/L)	599.7 ± 149.1	(451–749)	596.4 ± 134.0	(462–730)	609.0 ± 209.3	(400–818)	0.916
Proteins							
TOT PROT (g/dL)	7.0 ± 0.5	(6.4–7.5)	7.0 ± 0.5	(6.5–7.5)	6.9 ± 0.5	(6.4–7.5)	0.772
ALB (g/dL)	3.9 ± 0.5	(3.5–4.4)	4.0 ± 0.4	(3.5–4.4)	3.9 ± 0.5	(3.4–4.4)	0.746
GLOB (g/dL)	2.9 ± 0.4	(2.5–3.3)	2.9 ± 0.4	(2.4–3.3)	2.9 ± 0.4	(2.4–3.3)	0.998
ALB:GLOB	1.4 ± 0.3	(1.1–1.7)	1.4 ± 0.3	(1.1–1.7)	1.4 ± 0.3	(1.1–1.7)	0.682
Electrolytes							
NA (mmol/L)	151.0 ± 5.5	(146–157)	151.0 ± 5.7	(145–157)	151.0 ± 5.4	(146–156)	0.956
Cl^−^ (mmol/L)	99.6 ± 6.3	(93–106)	99.2 ± 5.5	(94–105)	99.9 ± 6.9	(93–107)	0.669
K (mmol/L)	5.3 ± 0.7	(4.6–5.9)	5.3 ± 0.6	(4.8–5.9)	5.3 ± 0.7	(4.5–6.0)	0.713
PO_4_ (mg/dL)	5.9 ± 1.1	(4.8–7.0)	5.9 ± 1.1	(4.8–7.0)	5.9 ± 1.2	(4.7–7.0)	0.921
Ca (mg/dL)	9.8 ± 0.7	(9.2–11)	9.9 ± 0.6	(9.3–10)	9.8 ± 0.7	(9.1–11)	0.676
CO_2_ (mmol/L)	26.7 ± 9.5	(17–36)	26.9 ± 8.4	(19–35)	26.5 ± 10.5	(16–37)	0.863
AG (mmol/L)	27.9 ± 10.6	(17–39)	27.8 ± 9.2	(19–37)	28.0 ± 11.7	(16–40)	0.997

**Table 6:** Hematology reference intervals for West Indian manatees from Puerto Rico, Guyana, Mexico, Belize, Brazil, and Florida. Puerto Rico values included all samples (calves, subadults, and adults). Abbreviations for parameters are detailed in the materials and methods section. Columns with an asterisk (^*∗*^) signify that the range values are minimum and maximum.

Parameter	Antillean manatees					Florida manatees	
	Puerto Rico	Guyana [7]	Mexico [8]	Belize [9]	Brazil [14]^*∗*^	Florida [6]	Florida [25]^*∗*^
	*n* = 70	*n* = 11	*n* = 18	*n* = 82	*n* = 30	*n* = 23	*n* = 52
WBC (10^9^/L)	4.0–8.0	4.6–8.6	3.9–9.1	3.4–7.9	4.4–11	4–12	2.8–14
RBC (10^6^/mm^3^)	2.1–3.3	2.2–2.8	2.3–3.3	2.2–3.0	2.5–3.0	2.4–3.4	2.2–3.4
Hb (g/dl)	9.0–14	8.9–11	9.8–13	9.5–12	9.1–11	9.8–13	9.4–14
HCT (%)	28–41	17–24	31–42	30–37	29–34	30–40	29–44
PLTS (10^3^/mm^3^)	207–390	—	138–266	156–384	—	195–412	111–424
Red blood cell indices							
MCV (fL)	120–137	—	—	100–146	109–116	122–149	114–140
MCH (pg)	40–43	—	—	36–44	33–38	38–46	37–45
MCHC (g/dL)	31–34	—	30–33	29–35	29–33	30–33	28–35
RDW (%)	15–21	—	—	—	—	—	14–23
White blood cell differential							
LYMPH (%)	28–57	—	—	—	—	—	—
MONO (%)	1–6	—	—	—	—	—	—
EOSIN (%)	0–3	—	—	—	—	0–0	—
BASO (%)	0–1	0–0	—	—	0–0	0–0	—
HET (%)	38–67	—	—	—	—	—	—

**Table 7:** Serum chemistry reference intervals for West Indian manatees from Puerto Rico, Florida, Guyana, Mexico, Belize, and Brazil. Puerto Rico values included all samples (calves, subadults, and adults). Abbreviations for parameters are detailed in the Materials and Methods section. Columns with an asterisk (^*∗*^) signify that the range values are minimum and maximum.

Parameter	Antillean manatees					Florida manatees	
	Puerto Rico	Guyana [7]	Mexico [8]	Belize [9]	Brazil [14]^*∗*^	Florida [6]	Florida [24]^*∗*^
	*n* = 70	*n* = 11	*n* = 18	*n* = 82	*n* = 30	*n* = 23	*n* = 55
Liver-associated enzymes and pigments							
LDH (U/L)	261–590	—	—	—	—	94–372	—
T BILI (mg/dL)	0.1–0.3	0.2–0.4	0.0–0.4	—	0–0.1	0–0.1	0–0.3
Muscle-associated enzymes							
ALT (U/L)	8.3–23	—	14–24	4.4–33	3.0–9.0	6.0–30	5.0–48
AST (U/L)	6.1–17	18–19	13–80	19–52	6.0–13	5.0–28	4.0–26
ALP (U/L)	58–99	45–80	—	52–106	214–412	64–183	39–192
CPK (U/L)	68–132	75–228	78–191	—	—	79–302	51–2966
Kidney-associated compounds and products							
BUN (mg/dL)	2.1–6.3	1.6–6.4	3.1–13	1.7–9.5	—	6.4–16	1.1–12
CREA (mg/dL)	1.1–1.9	1.0–1.4	0.0–4.4	1.0–2.4	1.5–2.3	0.4–2.1	0.6–3.8
BUN:CREA	1.4–2.8	—	—	—	—	—	—
UA (mg/dL)	0.4–1.4	—	—	—	1.6–2.2	—	—
Sugars, lipids, and pancreatic-associated enzymes							
GLU (mg/dL)	73–110	70–97	67–101	44–120	66–129	56–117	41–178
TRIG (mg/dL)	84–135	—	82–134	50–131	—	—	27–195
CHOL (mg/dL)	92–143	—	134–210	88–170	148–243	107–328	81–282
AMY (U/L)	451–749	—	—	—	—	—	—
Proteins							
T PROT (g/dL)	6.4–7.5	6.5–7.3	6.9–8.3	6.3–7.8	6.4–7.8	6.2–8.6	6.4–9.0
ALB (g/dL)	3.5–4.4	4.1–5.1	4.0–5.6	3.3–5.1	4.6–6.7	3.6–5.9	2.5–4.6
GLOB (g/dL)	2.5–3.3						
ALB:GLOB	1.1–1.7	—	1.9–3.7	—	—	—	0.6–1.3
Electrolytes							
NA (mmol/L)	146–157	138–149	—	142–160	—	142–157	143–158
CL (mmol/L)	93–106	92–105	—	87–105	—	90–103	78–106
K (mmol/L)	4.6–5.9	4.2–5.0	—	4.6–6.0	—	4.2–6.6	3.8–6.3
PO_4_ (mg/dL)	4.8–7.0	4.2–5.6	—	3.7–7.0	—	3.0–8.0	3.4–8.4
CA (mg/dL)	9.2–11	9.6–11	—	9.1–12	—	10–12	8.0–15
CO_2_ (mmol/L)	17–36	13–18	—	—	—	—	4.0–41
AG (mmol/L)	17–39	30–37	—	—	—	—	15–59

## Data Availability

The datasets used and/or analyzed during the current study can be obtained from the corresponding author upon request.
